# Mitochondrial Genome Mutations Associated with Myocardial Infarction

**DOI:** 10.1155/2018/9749457

**Published:** 2018-02-18

**Authors:** Margarita A. Sazonova, Anastasia I. Ryzhkova, Vasily V. Sinyov, Elena V. Galitsyna, Alexandra A. Melnichenko, Natalya A. Demakova, Igor A. Sobenin, Tatiana P. Shkurat, Alexander N. Orekhov

**Affiliations:** ^1^Laboratory of Angiopathology, Institute of General Pathology and Pathophysiology, Moscow 125315, Russia; ^2^Laboratory of Medical Genetics, National Medical Research Center of Cardiology, Moscow 121552, Russia; ^3^Department of Genetics, Southern Federal University, Rostov-on-Don 344006, Russia; ^4^Institute for Atherosclerosis Research, Skolkovo Innovation Center, Moscow 121609, Russia

## Abstract

Myocardial infarction is one of the clinical manifestations of coronary heart disease. In some cases, the cause of myocardial infarction may be atherosclerotic plaques which occurred in the human aorta. The association of mtDNA mutations with atherosclerotic lesions in human arteries was previously detected by our research group. In this study, we used samples of white blood cells collected from 225 patients with myocardial infarction and 239 control persons with no health complaints. DNA was isolated from the blood leukocyte samples. Then, PCR fragments of DNA were obtained. They contained the investigated regions of 11 mitochondrial genome mutations (m.5178C>A, m.3336T>C, m.652delG, m.12315G>A, m.14459G>A, m.652insG, m.14846G>A, m.13513G>A, m.1555A>G, m.15059G>A, m.3256C>T). According to the obtained results, three mutations of the human mitochondrial genome correlated with myocardial infarction. A positive correlation was observed for mutation m.5178C>A. At the same time, a highly significant negative correlation with myocardial infarction was observed for mutation m.14846G>A. One single-nucleotide substitution of m.12315G>A had a trend towards negative correlation. These mutations can potentially be useful for creating molecular/cellular models for studying the mechanisms of myocardial infarction and designing novel therapies. Moreover, these mutations can possibly be used for diagnostic purposes.

## 1. Introduction

Myocardial infarction is one of the clinical manifestations of coronary heart disease. In this serious disease, some myocardial contractile cells die. Subsequently, these cells are replaced by connective tissue. The death of cells is a consequence of coronary heart disease. At the same time, metabolism disturbance occurs and irreversible changes in cells develop [1]. In most cases, acute myocardial infarction occurs due to coronary artery thrombosis in the area of an atherosclerotic plaque [1, 2]. In particular, myocardial infarction can occur in patients with atherosclerosis, arterial hypertension, and coronary heart disease. The prime risk factors for developing MI are obesity, lack of motor performance, and smoking. The clinical picture of MI is distinguished by a great variety. That is why it is difficult to make the right diagnosis. The patient may have chest discomfort or irregular heartbeat. Sometimes there is a complete absence of pain. With atypical cases of myocardial infarction, there is abdominal pain, laborious breathing, or dyspnea [3, 4].

At the present time, there are no reliable algorithms for the early prognosis of myocardial infarction, which would determine an increased individual predisposition to this disease and its risk factor, atherosclerosis. The focus of further researches for the determination of the causes of myocardial infarction development should be transferred to the interaction of environmental, ecological, and molecular-genetic risk factors, as well as to the search of new methods and approaches to early diagnosis of individual predisposition. Molecular-genetic factors predisposing to the development of myocardial infarction have not been studied enough.

In a number of published articles, there has been a report of mutations and polymorphisms of the nuclear genome associated with a risk factor for atherosclerosis, such as myocardial infarction [5–9]. However, these data do not cover the full range of variability of myocardial infarction clinical manifestations.

The results obtained by our group suggest, however, that mitochondrial genome mutations can also be associated with myocardial infarction. We have previously demonstrated that mtDNA mutations were present in cells from atherosclerotic aortas and arteries [10–14]. In this work, we evaluated the association of these mutations with myocardial infarction as a risk factor for atherosclerosis.

Unlike nuclear genome mutations, in the analysis of mitochondrial genomes, there occurs not the determination of homo- and heterozygotes by mutation, but the detection of the heteroplasmy level of mtDNA mutations (the ratio of mutant copies of the mitochondrial genome to the total number of DNA molecules in the mitochondria). The method of determining the heteroplasmy level of mitochondrial genome mutations based on pyrosequencing technology was previously developed by our laboratory scientists [10, 15–18]. It should be noted that the developed method is the most accurate in assessing the heteroplasmy level of mitochondrial genome mutations [10, 15]. In pyrosequencing, a short DNA fragment (6–10 bp) containing the investigated mutation is studied [19, 20]. Therefore, the probability of mistakes in determining the heteroplasmy level of mutations is minimal [21–23].

## 2. Materials and Methods

A total of 464 subjects were enrolled in the Cardiology Research Complex MH RF and Moscow State University clinic. All study participants were aged between 40 and 55 years. The investigated sample included 225 patients with myocardial infarction and 239 control persons with no health complaints. The work was conducted in accordance with the Declaration of Helsinki. The study protocol has been approved by the Ethics Community of Cardiology Research Complex MH RF, and all subjects gave written informed consent upon enrollment.

DNA was isolated from the blood leukocyte samples of study participants. The phenol-chloroform extraction method, developed by the authors of the article [24–26] based on the Maniatis technology [27], was used.

Then, PCR fragments of DNA were obtained, which contained the investigated regions of 11 mitochondrial genome mutations (m.5178C>A, m.3336T>C, m.652delG, m.12315G>A, m.14459G>A, m.652insG, m.14846G>A, m.13513G>A, m.1555A>G, m.15059G>A, m.3256C>T).

Primers for PCR and the size of PCR fragments are listed in Table 1 [10].

Biotinylation of one of the primers for PCR was carried out with the aim of pyrosequencing the amplicon.

Each 30 *μ*l PCR reaction contained 0.4–0.6 *μ*g mitochondrial DNA, 16.6 *μ*M (NH_4_)_2_SO_4_, 0.3 pM of each primer, 200 *μ*M of each deoxyribonucleotriphosphate, 67 mM Tris-HCl (pH 8.8), MgCl_2_ (1.5 mM for m.14846G>A, m.15059G>A, and m.14459G>A; 2.5 mM for the rest of the investigated mutations), and 3 units of *Taq* polymerase [10].

In PCR, the following annealing temperatures were used for the primers [10]:

1. For mutations m.5178C>A, m.652delG, and m.652insG—60°C
2. For mutations m.3336T>C, m.14846G>A, m.13513G>A, m.15059G>A, and m.3256C>T—55°C
3. For mutations m.12315G>A, m.14459G>A, and m.1555A>G—50°C

As an apparatus for PCR, “PTC DNA Engine 200” was used.

The association of these mutations with atherosclerotic lesions in human arteries was previously established [10–13, 17]. The PCR fragments were analyzed on the automated pyrosequencing device PSQTMHS96MA (Biotage, Sweden) to determine the heteroplasmy level of mtDNA mutations [10].

Primers for pyrosequencing are listed in Table 2 [10].

The results were analyzed using the software package SPSS 22.0 [28]. Bootstrap analysis was used. Correlation was considered statistically significant at the level of *p* ≤ 0.05. The results at the significance level of *p* ≤ 0.2 were considered to show a tendency toward statistical significance.

## 3. Results and Discussion

For all the study participants, age and demographic characteristics were determined (Tables 3 and 4). The data in Table 4 is presented as an average value with the standard deviation indicated (in parentheses).

According to Table 3, the age of conventionally healthy participants ranged from 29 to 75 years. At the same time, the age of patients with myocardial infarction ranged from 43 to 87 years. The average age of conventionally healthy study participants was 13 years less than the age of patients with myocardial infarction.

It is noteworthy that women predominated in the group of conventionally healthy study participants. At the same time, men predominated in the group of patients with myocardial infarction.

Significant differences between conventionally healthy study participants and patients with myocardial infarction were found only for risk factors such as sex and age (Table 4). It is worth mentioning that the tendency to the occurrence of such differences was found for smoking frequency. Perhaps, by increasing the sample, these differences will become reliable.

For the present investigation, the 11 mitochondrial genome mutations were taken, for which, in preliminary studies, a connection with atherosclerosis was found [10–12, 17, 18]. First, we examined 42 mtDNA mutations, for which an association with various pathologies was found [10]. We investigated lipofibrous plaques and areas of normal aortic intima. Therewith, 11 mitochondrial genome mutations associated with atherosclerosis were detected. It was decided to investigate the identified mutations in a sample of patients with myocardial infarction.

An evaluation of Spearman correlation of the investigated mtDNA mutations with myocardial infarction is presented in Table 5.

The coefficient of correlation was necessary for us to identify the direction of linkage of mtDNA mutations with myocardial infarction. If the connection was positive, the mutations were associated with myocardial infarction. If it was negative, mutations showed an antipathological effect. A positive correlation was observed for mutation m.5178C>A. At the same time, a highly significant negative correlation with myocardial infarction was observed for mutation m.14846G>A. One single-nucleotide substitution of m.12315G>A had a trend towards negative correlation (*p* ≤ 0.1).

For the found three mitochondrial genome mutations, an analysis of the odds ratio to be associated with the occurrence of myocardial infarction or to have a protective effect from this pathology was made. According to the obtained data, the probability of the occurrence of myocardial infarction in carriers of the mitochondrial genome mutation m.5178C>A was 2.8-fold higher than that in the study participants in which this mutation is absent. At the same time, the probability of the occurrence of this pathology in carriers of mutation m.14846G>A and in carriers of mutation m.12315G>A was 2.4-fold lower and 1.15-fold lower, respectively, than that in the study participants without these mutations.

Therefore, the mtDNA mutation m.5178C>A was a risk factor for the occurrence of myocardial infarction. At the same time, mutations m.14846G>A and m.12315G>A had a protective effect concerning this pathology.

The three mutations that had a positive or negative correlation with myocardial infarction were located in the coding region of the mitochondrial genome. Mutations m.5178C>A and m.14846G>A were localized in the genes encoding the second protein subunit of NADH dehydrogenase and cytochrome B, respectively. Mutations in these genes can therefore lead to mitochondrial respiratory chain enzyme dysfunction. Mutation m.12315G>A was localized in the transport RNA gene Leu (recognition codon CUN). It can possibly lead to defects in the transport RNA and can affect protein synthesis, which, in turn, may result in deficiencies of mitochondrial respiratory chain enzymes. Therefore, the described mutations can eventually lead to energy deficiency in affected cells, which may play a role in pathological processes, including myocardial infarction.

It is necessary to note that for this research, we used the method of quantitative assessment of the heteroplasmy level of mtDNA mutations, developed by us on the basis of pyrosequencing technologies in 2007 [10, 18, 29]. Based on the threshold heteroplasmy level of the mutation, associated with myocardial infarction, we detected the significance of the differences in this parameter between patients with myocardial infarction and conventionally healthy participants in the study. At the same time, two groups of scientists from Japan investigated the frequency of the occurrence of m.5178C>A in the Japanese sample of patients and in healthy people using polymerase chain reaction-restriction fragment length polymorphism (PCR-RFLP) analysis with the restriction enzyme *Alu*I [30, 31]. They found that the frequency of occurrence of the 5178C allele is higher in the group of patients with myocardial infarction than in healthy people. Unfortunately, it is impossible to determine the heteroplasmy level of mutation, using the PCR-RFLP method. In consequence of this, Japanese scientists could not take into account patients who have a not very high threshold heteroplasmy level of mutation m.5178C>A linked to myocardial infarction.

According to a generally accepted opinion of scientists around the world, polymorphisms do not lead to pathologies, unlike mutations. The mtDNA mutation m.5178C>A, according to our data, was associated with atherosclerosis [11, 17]. In the present investigation, we have found a link of this mutation with myocardial infarction. Therefore, as a pathological variant of the mitochondrial genome mutation m.5178C>A, in our articles, we name it a “mutation” and not a polymorphism.

It is noteworthy that mutation m.14846G>A, according to the literature, leading to a progressive exercise of intolerance, proximal limb weakness, and attacks of myoglobinuria, showed a protective effect on myocardial infarction at a high level of significance [32]. This can mean that the molecular mechanisms which lead to exercise intolerance, proximal limb weakness, and attacks of myoglobinuria protect the heart from the occurrence of myocardial infarction.

According to data from the literature, mutation m.12315G>A turned out to be associated with mitochondrial myopathy, ophthalmoplegia, ptosis, limb weakness, sensorineural hearing loss, and pigmentary retinopathy [33, 34]. At the same time, in our study, m.12315G>A showed a tendency to have a protective effect on myocardial infarction. It can also indicate that the molecular mechanisms which lead to the occurrence and development of mitochondrial myopathy, ophthalmoplegia, ptosis, limb weakness, sensorineural hearing loss, and pigmentary retinopathy protect from myocardial infarction.

It may also be suggested that the differences between the Russian and the Japanese samples are connected with undersampling. We plan to expand our sample.

It is necessary to note, for a number of diseases, for example, cystic fibrosis, that a gradient in the spread of some mutations from west to east has been found. Supposedly in this case, we are dealing with a similar gradient in the spread of some mitochondrial genome mutations. This is confirmed by the fact that the two articles in which it is stated that mutation m.5178C>A is associated with a lower frequency of its occurrence in patients with myocardial infarction, compared to healthy people, belong to Japanese research groups [30, 31].

To answer this question, we plan to get in our further studies, with an increase in the size of our sample. Perhaps, with mutation m.12315G>A, we will get very significant differences between patients with myocardial infarction and conventionally healthy study participants.

## 4. Conclusion

In the present study, we report on three mutations of the human mitochondrial genome that correlated with myocardial infarction. A positive correlation was observed for mutation m.5178C>A. At the same time, a highly significant negative correlation with myocardial infarction was observed for mutation m.14846G>A. One single-nucleotide substitution of m.12315G>A had a trend towards negative correlation (*p* ≤ 0.1).

Therefore, the mtDNA mutation m.5178C>A was a risk factor for the occurrence of myocardial infarction. At the same time, mutations m.14846G>A and m.12315G>A had a protective effect concerning this pathology.

These mutations can potentially be useful for creating molecular/cellular models for studying the mechanisms of myocardial infarction and designing novel therapies. Moreover, these mutations can possibly be used for diagnostic purposes.

## Figures and Tables

**Table 1:** Primers for PCR.

Mutation	Primers	Size of PCR fragment
m.5178C>A	F: bio-GCAGTTGAGGTGGATTAAAC (4963–4982) R: GGAGTAGATTAGGCGTAGGTAG (5366–5345)	383 bp

m.3336T>C	F: bio-AGGACAAGAGAAATAAGGCC (3129–3149) R: ACGTTGGGGCCTTTGCGTAG (3422–3403)	294 bp

m.652delG	F: TAGACGGGCTCACATCAC (621–638) R: bio-GGGGTATCTAATCCCAGTTTGGGT (1087–1064)	467 bp

m.12315G>A	F: bio-CTCATGCCCCCATGTCTAA (12230–12249) R: TTACTTTTATTTGGAGTTGCAC (12337–12317)	108 bp

m.14459G>A	F: CAGCTTCCTACACTATTAAAGT (14303–14334) R: bio-GTTTTTTTAATTTATTTAGGGGG (14511–14489)	209 bp

m.652insG	F: TAGACGGGCTCACATCAC (621–638) R: bio-GGGGTATCTAATCCCAGTTTGGGT (1087–1064)	467 bp

m.14846G>A	F: bio-CATTATTCTCGCACGGACT (14671–14689) R: GCTATAGTTGCAAGCAGGAG (15120–15100)	450 bp

m.13513G>A	F: CCTCACAGGTTTCTACTCCAAA (13491–13512) R: bio-AAGTCCTAGGAAAGTGACAGCGAGG (13825–13806)	335 bp

m.1555A>G	F: TAGGTCAAGGTGTAGCCCATGAGGTGGCAA (1326–1355) R: bio-GTAAGGTGGAGTGGGTTTGGG (1704–1684)	379 bp

m.15059G>A	F: bio-CATTATTCTCGCACGGACT (14671–14689) R: GCTATAGTTGCAAGCAGGAG (15120–15100)	450 bp

m.3256C>T	F: bio-AGGACAAGAGAAATAAGGCC (3129–3149) R: ACGTTGGGGCCTTTGCGTAG (3422–3403)	294 bp

**Table 2:** Primers for pyrosequencing.

Mutation	Primer
m.5178C>A	ATTAAGGGTGTTAGTCATGT (5200–5181)
m.3336T>C	TGCGATTAGAATGGGTAC (3354–3337)
m.652delG	CCCATAAACAAATA (639–651)
m.12315G>A	TTTGGAGTTGCAC (12328–12316)
m.14459G>A	GATACTCCTCAATAGCCA (14439–14456)
m.652insG	CCCATAAACAAATA (639–651)
m.14846G>A	GCGCCAAGGAGTGA (14861–14848)
m.13513G>A	AGGTTTCTACTCCAA (13497–13511)
m.1555A>G	ACGCATTTATATAGAGGA (1537–1554)
m.15059G>A	TTTCTGAGTAGAGAAATGAT (15080–15061)
m.3256C>T	AAGAAGAGGAATTGA (3300–3286)

**Table 3:** Age characteristics of the study participants.

Study participants	Age			Standard deviation
	Minimum (years)	Mean (years)	Maximum (years)	
Conventionally healthy	29	52	75	8.5
Patients with myocardial infarction	43	65	87	8.3

**Table 4:** Demographic characteristics of the study participants.

Parameter	Conventionally healthy study participants	Patients with myocardial infarction	Significance of differences
Sex, m/f	109 : 130	135 : 90	0.008^∗^
Age, years	52 (8.5)	65 (8.3)	0.027^∗^
Body mass index, kg/m^2^	23.5 (4.3)	29.1 (5.2)	0.43
Systolic blood pressure, mmHg	123 (19)	142 (25)	0.21
Diastolic blood pressure, mmHg	81 (15)	87 (23)	0.35
Smoking, %	19	41	0.12

^∗^Significant differences between conventionally healthy study participants and patients with myocardial infarction.

**Table 5:** Spearman correlation of 11 mtDNA mutations with myocardial infarction.

Mutation	Correlation coefficient	Significance
m.5178C>A	0.109	0.045^∗∗^
m.3336T>C	0.051	0.198
m.652delG	0.053	0.242
m.12315G>A	−0.096	0.065^∗^
m.14459G>A	0.064	0.187
m.652insG	−0.045	0.229
m.13513G>A	0.069	1.174
m.14846G>A	−0.127	0.001^∗∗^
m.1555A>G	−0.059	0.191
m.15059G>A	0.079	0.116
m.3256C>T	0.075	0.111

^∗∗^ *p* ≤ 0.05; ^∗^*p* ≤ 0.1.
